# The rat adequately reflects human responses to exercise in blood biochemical profile: a comparative study

**DOI:** 10.14814/phy2.12293

**Published:** 2015-02-12

**Authors:** Georgios Goutianos, Aikaterini Tzioura, Antonios Kyparos, Vassilis Paschalis, Nikos V Margaritelis, Aristidis S Veskoukis, Andreas Zafeiridis, Konstantina Dipla, Michalis G Nikolaidis, Ioannis S Vrabas

**Affiliations:** 1 Exercise Physiology and Biochemistry Laboratory, Department of Physical Education and Sports Science at Serres, Aristotle University of ThessalonikiSerres, Greece; 2 Department of Hematology – Blood Bank, General Hospital of SerresSerres, Greece; 3 Department of Physical Education and Sport Science, University of ThessalyKaries, Trikala, Greece

**Keywords:** Animal models, biomarkers, blood, exercise, human, rat

## Abstract

Animal models are widely used in biology and the findings of animal research are traditionally projected to humans. However, recent publications have raised concerns with regard to what extent animals and humans respond similar to physiological stimuli. Original data on direct in vivo comparison between animals and humans are scarce and no study has addressed this issue after exercise. We aimed to compare side by side in the same experimental setup rat and human responses to an acute exercise bout of matched intensity and duration. Rats and humans ran on a treadmill at 86% of maximal velocity until exhaustion. Pre and post exercise we measured 30 blood chemistry parameters, which evaluate iron status, lipid profile, glucose regulation, protein metabolism, liver, and renal function. ANOVA indicated that almost all biochemical parameters followed a similar alteration pattern post exercise in rats and humans. In fact, there were only 2/30 significant species × exercise interactions (in testosterone and globulins), indicating different responses to exercise between rats and humans. On the contrary, the main effect of exercise was significant in 15/30 parameters and marginally nonsignificant in other two parameters (copper, *P* = 0.060 and apolipoprotein B, *P* = 0.058). Our major finding is that the rat adequately mimics human responses to exercise in those basic blood biochemical parameters reported here. The physiological resemblance of rat and human blood responses after exercise to exhaustion on a treadmill indicates that the use of blood chemistry in rats for exercise physiology research is justified.

## Introduction

Animal models, particularly rodents, are widely used in biological sciences and the findings of animal research are traditionally projected to humans. However, recent publications have raised serious concerns with regard to what extent rodents and humans respond similar to several physiological stimuli, thus, challenging the intuitively accepted notion that data acquired in animal settings are transferable and applicable to humans in vivo. The recently reanimated debate over the appropriateness of rodent use in biological research is mainly based on opinion letters (Iannaccone and Jacob 2009; Rice 2012; Cauwels et al. 2013; Leist and Hartung 2013; Osterburg et al. 2013; Tompkins et al. 2013; Warren et al. 2013) and review papers (Hackam and Redelmeier 2006; Knight 2007; Perel et al. 2007; Rittirsch et al. 2007; Rennie et al. 2010; Wang et al. 2010; van der Worp et al. 2010; Greek et al. 2012; Rubio-Aliaga 2012).

Beyond the above-mentioned review papers, original data on direct in vivo comparison between healthy animals and humans are scarce and – to the best of our knowledge – are limited to inflammatory disease (Seok et al. 2013) and calorie restriction (Mercken et al. 2013). However, even these two in vivo studies that have directly compared rodents and humans response under the same stimulus are contradictory. In fact, it has been reported that gene expression alterations to various inflammatory etiologies correlate poorly between mice and human models (Seok et al. 2013), suggesting that mouse may not be an appropriate model to study inflammation in humans. On the contrary, Mercken et al. (2013) has demonstrated that the overall physiological, molecular, and inflammatory response to long-term caloric restriction was similar in rats and humans.

In biomedical research, the most frequently used animal experimentation model is rodent. In exercise physiology research, excluding transgenic animals, the use of rats probably surpasses that of mice. The major advantage of rats over mice is the greater tissue sample size and the larger blood volume that allows measurement of a complete set of blood biomarkers. Rats have been used to address diverse research questions in the exercise physiology field including physical performance (Veskoukis et al. 2008; Booth and Laye 2009), metabolism (Nikolaidis et al. 2004; Brooks 2009), fatigue (Allen et al. 2008; Kyparos et al. 2012), muscle atrophy (Fitts et al. 2001; Kyparos et al. 2005), gene expression, and signaling pathways (Nikolaidis et al. 2003; Röckl et al. 2008).

Thus, it becomes apparent that direct and well controlled in vivo comparisons between rats and humans are currently required to shed light on whether rat is an appropriate model for studying human responses to exercise. Therefore, the aim of this study was to address this issue by directly comparing side by side in the same experimental setup rat and human responses to an acute submaximal exercise bout to exhaustion of matched intensity and duration on a treadmill. We measured a wide range of blood chemistry parameters, which are linked to various organs, tissues, functions, and diseases. The diversity and extent of the biochemical measurements facilitates our goal to draw a holistic picture of how rat and human respond to an identical exercise stimulus. We anticipate that the outcome of this holistic approach will be useful not only for exercise biologists but for all biomedical scientists.

## Materials and Methods

### Rat study

#### Rats

Forty adult male Wistar rats (*Rattus norvegicus)*, aged 11–12 weeks old and weighing 294–428 g (363 ± 31 g) were used in the study. The animals were housed under a 12 h light: 12 h dark cycle, controlled temperature (21–23°C) and controlled humidity (50–70%). Commercial rat chow and tap water were provided ad libitum. All procedures were in accordance with the European Union guidelines for the care and use of laboratory animals, as well as the “Principles of laboratory animal care” (NIH publication No. 86-23, revised 1985). The project was reviewed and approved by the institutional review board and the appropriate state authority.

#### Familiarization to treadmill running

Rats were randomly divided into an ambulatory control group or an exercise group. All rats were familiarized with running on a motor-driven treadmill based on Koch et al. (2013). Briefly, the first 2 days the rats were just placed on the horizontally moving treadmill belt (10 m/min). Rats moving backwards were repositioned forward by the researchers. In the next 2 days the rats were trained to understand that failure to run results to contact with the electrical grid at the back of the treadmill. Thereafter, for the next 7 days the rats were running incrementally from 10 to 40 m/min (2.5 min at 10 m/min, 2.5 min at 20 m/min, 2.5 min at 30 m/min, 2.5 min at 40 m/min) for 10 min/day. Sedentary rats also were familiarized to treadmill running using the same protocol. The rats not completing this familiarization procedure were excluded from the study.

#### Incremental exercise test for determination of maximal velocity

Following a 5-min warm-up, initial speed on the horizontal treadmill was set at 12 m/min. Speed was thereafter increased by 4.2 m/min every minute. Maximal velocity was determined as the velocity maintained for at least 1 min. A similar to our approach has been previously used by other researchers (Copp et al. 2010). Criteria for exhaustion were as follows: conduct with the electrical grid for third time (Koch et al. 2013) and the loss of the righting reflex when the rats were laid on their backs. When necessary for rats to run, their tails were stimulated using a soft bristle brush.

#### Exercise protocol

The rats ran horizontally on a treadmill at a speed corresponding to 86% of maximal running velocity. After a 5-min warm-up, the protocol started at a speed of 20 m/min. Initial speed was gradually increased to reach the designated speed in ∼30 sec. Time to exhaustion at 86% of maximal velocity was determined as the time elapsed between achievement of the designated speed and exercise termination. Criteria for exhaustion were the same, as those of the incremental exercise test for determination of maximal running velocity. All exercise protocols were supervised by the same experienced researcher. Food was removed from the cages 8 h earlier, to minimize the influence of the last feeding on the biochemical parameters. Blood samples were collected at rest for the ambulatory control group and immediately after exercise for the exercise group. Due to technical failures, blood sampling was not accomplished in four rats from the ambulatory group and in two rats from the exercise group.

### Human study

#### Humans

Thirty male, nonsmokers, recreationally active humans participated in this study (age, 21.1 ± 3.1 years; weight, 73.5 ± 8.4 kg; height, 180 ± 6 cm). Participants received detailed information about the possible risks and discomforts associated with the experimental procedure. They signed a health screening questionnaire and gave written informed consent to participate in the study, which was conducted in accordance with the declaration of Helsinki of 1975, as revised in 2008 and was approved by the Institution's ethics committee.

#### Familiarization to treadmill running

Each participant visited the laboratory once and was familiarized with running on the treadmill (Runrace, Technogym, Gambettola, Italy) at a self-selected speed for 5 min. After that, the speed was gradually increased to 12 km/h for 1 min in all participants.

#### Incremental exercise test for determination of maximal volitional velocity

A week after the familiarization, participants performed an incremental exercise test to exhaustion for the determination of maximal volitional velocity (Nikolaidis et al. 2012). The test was conducted on the same treadmill between 9 and 11 am. After a 5-min warm-up, the speed was set at 10 km/h and was increased by 0.5 km/h every minute until exhaustion. Maximal volitional velocity was determined as the maximal velocity maintained for at least 1 min. Similar to our approach has been previously used by other researchers (Midgley et al. 2006). Criteria for exhaustion were a maximal heart rate value within age-predicted maximal value (220-age ± 10%) and participants’ inability to maintain the designated speed despite intense encouragement.

#### Exercise protocol

Participants ran horizontally on the same treadmill at a speed corresponding to 86% of maximal volitional velocity. After a 5-min warm-up, the test started at a speed of 10 km/h. Initial speed was gradually increased to reach the designated speed in ∼30 sec. Time to exhaustion at 86% of maximal volitional velocity was determined as the time elapsed between achievement of the designated speed and exercise termination. Criteria for exhaustion were the same, as those of the incremental exercise test for determination of maximal volitional velocity. Overnight fasting blood samples were collected at rest and after exercise.

### Blood collection and analysis

Rats were deeply anesthetized by exposure to ether. The depth of anesthesia was assured by the constriction of the pupils as well as simple sensory tests, such as the absence of eye blinking when the eyelid was touched and the absence of foot withdrawal when the foot was pinched. Then, the thoracic cavity was opened. Whole blood was collected in EDTA vacutainer tubes (BD Vacutainer Systems, Plymouth, UK), via cardiac puncture in the right ventricle using a 10 mL heparin-treated syringe (Terrumo, Tokyo, Japan). Whole blood was collected from humans in EDTA vacutainer tubes by venipuncture in a superficial arm vein. Whole blood samples of rats and humans were immediately centrifuged (1500 g, 4°C, 10 min) for separation of plasma from blood cells. Plasma samples were stored at −80°C for later analysis. All biochemical parameters were measured with Abbot Architect ci 8200 and Abbot Architect ci 16200 (Abbott Diagnostics, Abbot park, IL) automated clinical analyzers. The homeostasis model assessment (HOMA) was used as a surrogate measure of insulin resistance and was calculated as fasting insulin (*μ*IU/mL) × fasting glucose (mmol/L)/22.5. A point to mention is that certain variables measured in this study (e.g., vitamin D) do not provide much information on the nature of the exercise perturbation, whereas other variables although directly affected by acute exercise (e.g., lactate) were not measured for technical reasons.

### Plasma volume

Postexercise plasma volume changes were calculated on the basis of hematocrit (Hct) and hemoglobin (Hb) changes, using the method employed by Dill and Costill (1974). Hematocrit was measured by microcentrifugation and hemoglobin was measured using a kit from Spinreact (Santa Coloma, Spain).

### Statistical analysis

The distribution of all dependent variables was examined using the Shapiro–Wilk test and was found not to differ significantly from normality. Physical characteristics and performance parameters were analyzed by using unpaired Student's t-tests. A two way ANOVA (Species [rat or human] × Exercise [pre or post exercise]) was used to analyze all parameters measured in blood plasma. If a significant interaction was obtained, pairwise comparisons were performed through simple main effect analysis. Data are presented as mean ± SD and significance level was set at *P* < 0.05.

## Results

### Exercise

The maximal running velocity achieved in the incremental test on the treadmill was 3.75 ± 0.91 km/h (62.5 m/min) for rats and 15.5 ± 1.8 km/h for humans. Time to exhaustion at 86% of the maximal running velocity was 11.1 ± 1.5 min for rats and 12.2 ± 1.5 min for humans (mean ± SD). There were no significant differences in time to exhaustion between rats and humans (*P* > 0.05).

### Plasma volume

Hct and Hb did not change significantly (*P* > 0.05) from pre to postexercise in either rats (pre: 44.1 ± 1.2%, 15.1 ± 0.7 g/dL and post: 44.4 ± 1.1%, 14.9 ± 0.6%, for Hct and Hb, respectively) or humans (pre: 45.1 ± 1.7%, 14.1 ± 0.5 g/dL and post: 45.8 ± 1.6%, 14.2 ± 0.4%, for Hct and Hb, respectively). Similarly, no significant differences were observed in plasma volume from pre to post in both rats and humans. Postexercise plasma volume was 98 ± 3% and 99 ± 5% of pre-exercise plasma volume, for rats and humans, respectively.

### Blood biochemical profile

**Figure 1:**
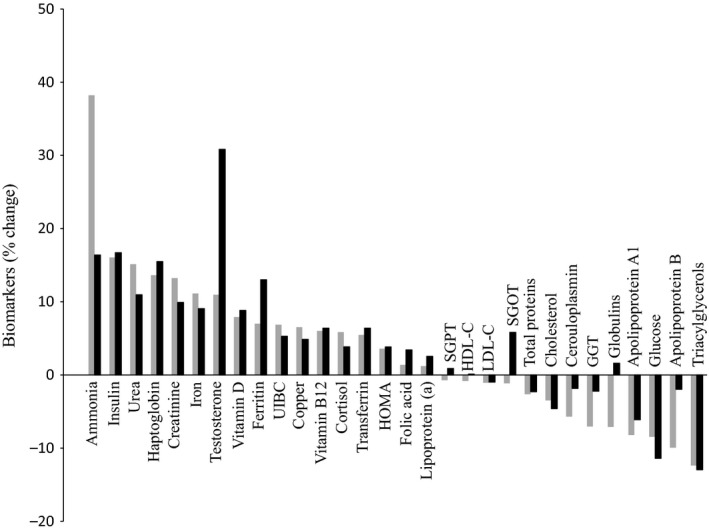
Percent changes in blood biochemical parameters after acute exhaustive exercise in rats (gray) and humans (black). GGT, gamma – glutamyl transferase; HDL-C, high-density lipoprotein cholesterol; HOMA, homeostasis model assessment; LDL-C, low-density lipoprotein cholesterol; SGOT, serum glutamic oxaloacetic transaminase; SGPT, serum glutamic – pyruvic transaminase; UIBC, unsaturated iron binding capacity.

Blood biochemical parameters pre and postexercise in rats and humans are presented in Table1. Postexercise percent changes in the blood biochemical parameters are presented in Figure1. In general, ANOVA indicated that almost all biochemical parameters followed a similar alteration pattern post exercise in rats and humans. In fact, there were only 2/30 significant species × exercise interactions (indicating different responses to exercise between rats and humans), one for testosterone and one for globulins. On the contrary, the main effect of exercise was significant in 15/30 parameters and marginally nonsignificant in other two parameters (copper, *P* = 0.060 and apolipoprotein B, *P* = 0.058). Furthermore, the main effect of species was significant in 22/30 parameters, indicating different levels of blood measures between rats and humans at rest.

**Table 1:** Blood plasma biochemical parameters pre and post exercise in rats and humans (mean ± SD).

Parameter	Pre	Post	S	E	S × E	Parameter	Pre	Post	S	E	S × E
Insulin (*μ*IU/mL)						Haptoglobin (mg/dL)					
Rat	13.3 ± 5.2	15.5 ± 6.7#	0.030	0.037	0.812	Rat	136 ± 58	155 ± 49#	0.074	0.003	0.615
Human	10.3 ± 2.7	12.0 ± 3.3*				Human	160 ± 39	184 ± 34*			
Glucose (mg/dL)						UIBC (*μ*g/dL)					
Rat	145 ± 25#	133 ± 20#*	<0.001	0.004	0.761	Rat	248 ± 56#	265 ± 57#	<0.001	0.011	0.871
Human	88.3 ± 7.0	78.3 ± 9.7*				Human	364 ± 21	383 ± 13*			
HOMA						Cerouloplasmin (mg/dL)					
Rat	4.38 ± 2.04#	4.54 ± 2.10#	<0.001	0.684	0.893	Rat	96.5 ± 36.5#	91.0 ± 31.1#	<0.001	0.590	0.665
Human	2.03 ± 0.59	2.11 ± 0.65				Human	32.5 ± 6.9	31.9 ± 6.7			
Testosterone (ng/dL)						Creatinine (mg/dL)					
Rat	517 ± 160	574 ± 164#	0.005	<0.001	0.006	Rat	1.51 ± 0.47#	1.71 ± 0.43#*	<0.001	0.012	0.096
Human	632 ± 214	887 ± 224*				Human	0.68 ± 0.18	0.75 ± 0.15*			
Cortisol (*μ*g/dL)						Ammonia (*μ*g/dL)					
Rat	15.8 ± 6.9#	16.7 ± 3.7#	0.004	0.299	0.715	Rat	16.2 ± 19.0	22.4 ± 20.9#	0.016	0.151	0.313
Human	11.6 ± 3.1	12.1 ± 3.1				Human	6.75 ± 6.71	7.86 ± 7.89			
Cholesterol (mg/dL)						Urea (mg/dL)					
Rat	234 ± 39#	226 ± 38#	0.001	0.004	0.897	Rat	15.2 ± 3.7#	17.5 ± 3.9#*	0.003	<0.001	0.881
Human	193 ± 24	184 ± 24*				Human	20.1 ± 5.3	22.3 ± 5.4*			
HDL-C (mg/dL)						GGT (IU/L)					
Rat	55.1 ± 6.9#	54.7 ± 6.6#	0.003	0.820	0.761	Rat	127 ± 64#	118 ± 60.#	<0.001	0.646	0.684
Human	63.9 ± 9.9	63.9 ± 9.8				Human	23.9 ± 13.0	22.6 ± 10.9			
LDL-C (mg/dL)						SGOT (IU/L)					
Rat	150 ± 17#	148 ± 20#	0.002	0.417	0.989	Rat	79.2 ± 19.0#	78.3 ± 18.8#	<0.001	0.971	0.740
Human	123 ± 26	122 ± 26				Human	12.6 ± 4.2	13.3 ± 4.4			
Triacylglycerols (mg/dL)						SGPT (IU/L)					
Rat	204 ± 68#	179 ± 48#*	<0.001	<0.001	0.464	Rat	219 ± 22#	217 ± 23#	<0.001	0.877	0.842
Human	140 ± 33	122 ± 30*				Human	22.7 ± 9.6	22.9 ± 10.1			
Apolipoprotein A1 (mg/dL)						Folic acid (ng/mL)					
Rat	169 ± 21#	155 ± 25#*	0.003	<0.001	0.316	Rat	7.88 ± 3.57#	7.99 ± 3.75#	<0.001	0.480	0.608
Human	144 ± 20	135 ± 18*				Human	19.7 ± 10.0	20.4 ± 10.1			
Apolipoprotein B (mg/dL)						Vitamin B12 (pg/mL)					
Rat	123 ± 28#	111 ± 23#*	<0.001	0.058	0.149	Rat	300 ± 66#	318 ± 76#	<0.001	0.005	0.233
Human	88.1 ± 11.6	86.4 ± 11.1*				Human	658 ± 143	700 ± 147*			
Lipoprotein (a) (mg/dL)						Copper (*μ*g/dL)					
Rat	25.1 ± 11.2	25.4 ± 10.8	0.174	0.615	0.918	Rat	119 ± 28#	126 ± 27#*	<0.001	0.060	0.560
Human	20.3 ± 9.5	20.8 ± 9.6				Human	83.8 ± 13.6	87.9 ± 13.3*			
Iron (*μ*g/dL)						Total proteins (g/dL)					
Rat	142 ± 46	157 ± 44	0.560	0.043	0.789	Rat	7.68 ± 0.49	7.47 ± 0.51	0.380	0.026	0.843
Human	135 ± 52	147 ± 53*				Human	7.55 ± 0.35	7.37 ± 0.38*			
Ferritin (ng/mL)						Globulins (g/dL)					
Rat	95.9 ± 53.5	103 ± 57#	0.081	0.048	0.350	Rat	3.62 ± 0.37	3.36 ± 0.40	0.319	0.149	0.031
Human	140 ± 102	159 ± 110*				Human	3.33 ± 0.42	3.39 ± 0.49			
Transferrin (mg/dL)						Vitamin D (ng/mL)					
Rat	278 ± 58	293 ± 51	0.239	0.019	0.783	Rat	9.61 ± 4.49	10.4 ± 4.8	0.141	0.340	0.899
Human	298 ± 57	317 ± 57*				Human	11.3 ± 3.9	12.3 ± 3.9			

HOMA, fasting glucose (*μ*IU/mL) × fasting glucose (mmol/L)/22.5; HDL-C, high-density lipoprotein cholesterol; LDL-C, low-density lipoprotein cholesterol; GGT, Gamma–glutamyltransferase; SGOT, serum glutamic oxaloacetic transaminase; SGPT, serum glutamic–pyruvic transaminase; UIBC, unsaturated iron binding capacity. S × E: 2-way interaction for species and exercise; S: Main effect of species; E: Main effect of exercise.

# Significant difference between Human and Rat in the same time point (*P *<* *0.05).

* Significant difference from preexercise (*P *<* *0.05).

## Discussion

To our knowledge, this is the first study which directly compared side by side in the same experimental setup rat and human blood biochemical profile responses to an acute submaximal exercise to exhaustion matched for intensity and duration on a treadmill. Our major finding is that the rat adequately mimics human responses to exercise in basic blood biochemical profile (i.e., iron status, lipid profile, glucose regulation, protein metabolism, liver and renal function). Acknowledging the limitations in all animal models (Cholewa et al. 2014; Seo et al. 2014), the physiological resemblance of rat and human blood responses after acute exercise to exhaustion on the treadmill indicates that the wide use of rats in exercise physiology research is justified in the blood parameters determined in this study.

The changes in blood biochemical parameters in response to exercise have similar qualitative and quantitative characteristics for both rats and humans. More specifically, 26/30 variables changed toward the same direction after exercise (qualitative agreement). Likewise, the magnitude of these uniform changes was also very similar (7.9% in rats and 7.3% in humans; quantitative agreement). Moreover, only two significant interactions were found between species and exercise (in testosterone and globulin), thus statistically verifying the similar pattern of blood responses following exercise in rats and humans. It is noteworthy that humans and rats responded qualitatively and quantitatively in a similar manner to exercise, despite the fact that there were noticeable differences in resting values in 19/30 parameters measured in blood. For example, even though rats had significantly higher resting triacylglycerol concentration compared to humans (204 mg/dL vs. 140 mg/dL, respectively), the percent decrease in triacylglycerol concentration following acute exercise was similar in rats (by 12.4%) and humans (by 13.0%). This illustrates that the initial absolute resting values of blood biomarkers do not predict the responses to exercise across species. It is worth mentioning that if outbred rats had been used (instead of the inbred rats used in this study) then it is likely that greater variability would have been observed, leading to disparate results between the two species. This may be an interesting theme for future research.

Noteworthy, the similarities in blood biochemical responses between rats and humans after acute exercise appeared despite the well-described physiological differences between the two species. For example, in this study lipid metabolism appears to be different between rodents and humans, as indicated by their differences in the resting levels of the relevant markers (e.g., LDL-C, LDL-C, triacylglycerols, cholesterol).

Other known physiological differences between the two species are the following: there are four myosin heavy chain isoforms in rat muscle, whereas only three in human muscle (Pellegrino et al. 2003), there are more fast fiber types and mitochondria in rat muscle than in human muscle (Schiaffino and Reggiani 2011), the speed of muscle contraction is greater in rats than in humans (Schiaffino and Reggiani 2011), the metabolic activity of skeletal muscle at rest is greater in rats than in humans (Schiaffino and Reggiani 2011), the energetic cost at a given exercise intensity is higher in rats compared to humans (Taylor et al. 1980), the heart rate, the respiratory rate, and oxygen consumption are higher in resting state in rats than in humans (Davies and Morris 1993) and, obviously, there are critical kinetic and kinematic differences between rats and humans during locomotion (Hosoido et al. 2013). The above further strengthens the role of exercise to act as a stimulus for challenging homeostasis of the body and exploring the potential differences or similarities in underlying mechanisms between species.

In this study, we chose to use treadmill running because it is the most extensively used exercise model (Wang et al. 2010) and allows complete control of the exercise workload (Seo et al. 2014), permitting thereby the comparison between rats and humans. To our knowledge, this is the first study that has directly examined rat and human responses after a matched exercise stimulus. We found two relevant studies in which rats and humans were directly compared after exercise, yet the exercise stimulus was not matched for both species. In one of these studies, rats and humans exercised for 120 min at similar intensity (65% and 60% of maximal oxygen uptake for rats and humans, respectively) but different exercise modality (humans cycled and rats ran on a treadmill) (Bradley et al. 2012). It was reported that muscle fatty acid translocase increased by 20% in rats and 75% in humans, whereas plasma membrane fatty acid-binding protein increased by 30% in rats and 20% in humans (Bradley et al. 2012), suggesting a partial agreement between rats and humans. In the second relevant study found in the literature, rats ran on a treadmill at a constant speed of 28 m/min until exhaustion (lasted 3.7 h), whereas humans ran outdoors for 16–19 km (the intensity was not reported) (Dohm et al. 1982). It was reported that urea and N-methylhistidine excretion in urine increased by 23% and 36%, respectively, in rats, whereas the respective changes of urea and N-methylhistidine were 18% and 23% in humans, suggesting a fairly good agreement of two protein catabolism markers in urine between rats and humans (Dohm et al. 1982). The data of these two studies along with the findings of this study, provide evidence to support that the rat is an appropriate animal model to mimic human changes in blood biochemical profile after exercise.

This study would have been more comprehensive if there were additional to the immediately postexercise sampling points, as the maximal response in some variables known to peak at later time points (e.g., max HDL-C peak in humans occurs 24 h post exercise) may have been missed. However, although this could be feasible in humans it would not be possible in rats, because in rats the blood was collected via cardiac puncture and the rats were killed at the time of blood sampling (exsanguination). Therefore, if more blood sampling points had been included, this would dramatically increase the number of rats required for each additional sampling point. Instead, the study was delimited to pre–post exercise sampling which are the “typical” sampling points in most exercise studies.

Exercise at 86% of maximal velocity and time to exhaustion of about 11–12 min in rats and humans demonstrate that both species relied on both aerobic and anaerobic mechanisms for energy provision. Although the approach of using a short high intensity exercise could have maximized inflammatory markers and other stress-related variables in blood, it would not be optimal for measuring the changes in blood lipids. Generally, maximal alterations in blood lipid levels after exercise are induced when energy expenditure is between 350 and 1150 kcal (Crouse et al. 1997; Ferguson et al. 1998), which typically requires 30–60 min of moderate exercise intensity. Nevertheless, even with the relatively short high intensity exercise scheme used in this study, there were significant pre–post exercise changes in cholesterol (humans), triacylglycerol (rats and humans), as well as apolipoprotein A1 and B (rats and humans).

Finally, it has been demonstrated that anesthetics – including ether – may affect some of the variables measured, for example, insulin and glucose (Aynsley-Green et al. 1973; Zardooz et al. 2010). However, we consider that the typical procedure followed by the experienced veterinary in controlling the dose of ether administered for anesthetizing the rats has minimized the potential effect of the anesthetic on the parameters measured and cancelled out its impact on animals.

## Conclusions

The rat model is a key element in advancing biological research. The prevailing assumption that the responses to exercise obtained from rat models mimic human responses to exercise is supported by our study at least regarding most of the blood parameters measured. Employing a side by side comparison and implementing a matched exercise stimulus for both species, we demonstrated that rat adequately reflected human responses to acute exercise in blood parameters linked to various organs, tissues, functions, and diseases. Although it is plausible to anticipate similar blood profile changes in humans and rats after multiple exercise sessions, differences between species following a period of training are not precluded. Our study highlights the effectiveness of exercise as a physiological environmental stimulus to elicit similar responses between two species at least in the variables assessed. It is vital that future research directly compares rat and human responses to acute and chronic exercise in additional variables and sampling points. In this regard, the use of exercise as a model to challenge homeostasis of the body for investigating biological phenomena, potential interventions, and underlying mechanisms in rats and humans is strengthened.
